# Etiologies of Long-Term Postcholecystectomy Symptoms: A Systematic Review

**DOI:** 10.1155/2019/4278373

**Published:** 2019-04-14

**Authors:** Carmen S. S. Latenstein, Sarah Z. Wennmacker, Judith J. de Jong, Cornelis J. H. M. van Laarhoven, Joost P. H. Drenth, Philip R. de Reuver

**Affiliations:** ^1^Department of Surgery, Radboud University Medical Centre, Nijmegen, Netherlands; ^2^Department of Gastroenterology, Radboud University Medical Centre, Nijmegen, Netherlands

## Abstract

**Background:**

Cholecystectomy does not relieve abdominal symptoms in up to 40% of patients. With 700,000 cholecystectomies performed in the US, annually, about 280,000 patients are left with symptoms, making this a serious problem. We performed a systematic review to determine the different etiologies of long-term postcholecystectomy symptoms with the aim to provide guidance for clinicians treating these patients.

**Methods:**

A systematic search of the literature was performed using MEDLINE, EMBASE, and Web of Science. Articles describing at least one possible etiology of long-term symptoms after a laparoscopic cholecystectomy were included in this review. Long-term symptoms were defined as abdominal symptoms that were present at least four weeks after cholecystectomy, either persistent or incident. The etiologies of persistent and incident symptoms after LC and the mechanism or hypothesis behind the etiologies are provided. If available, the prevalence of the discussed etiology is provided.

**Results:**

The search strategy identified 3320 articles of which 130 articles were included. Etiologies for persistent symptoms were residual and newly formed gallstones (41 studies, prevalence ranged from 0.2 to 23%), coexistent diseases (64 studies, prevalence 1-65%), and psychological distress (13 studies, no prevalence provided). Etiologies for incident symptoms were surgical complications (21 studies, prevalence 1-3%) and physiological changes (39 studies, prevalence 16-58%). Sphincter of Oddi dysfunction (SOD) was reported as an etiology for both persistent and incident symptoms (21 studies, prevalence 3-40%).

**Conclusion:**

Long-term postcholecystectomy symptoms vary amongst patients, arise from different etiologies, and require specific diagnostic and treatment strategies. Most symptoms after cholecystectomy seem to be caused by coexistent diseases and physiological changes due to cholecystectomy. The outcome of this research is summarized in a decision tree to give clinical guidance on the treatment of patients with symptoms after cholecystectomy.

## 1. Introduction

In the United States (US), approximately 1.8 million patients are diagnosed with gallstones every year [1]. In the majority of patients, gallstones will stay asymptomatic. Approximately 20% of patients will experience symptoms, like a biliary colic, for which laparoscopic cholecystectomy (LC) is the preferred treatment [2–4]. As a consequence, LC is one of the most performed elective abdominal surgeries worldwide, with approximately 700,000 LCs in the US [5].

Although LC is the preferred treatment to relieve symptoms, previous studies show that long-term abdominal symptoms are present in up to 40% of patients after LC [6–9]. This equals a yearly growth of 280,000 cases with abdominal symptoms after LC in the US. Patients suffer from symptoms like diarrhea, gas bloating, nausea, vomiting, jaundice, or abdominal pain. These symptoms after LC are a significant burden to health care systems, as 56% of patients need additional health care for diagnosis and treatment, against direct median hospital costs of $555 per year per patient. Moreover, sick leave and production loss of employed patients add an additional $361 per year per patient for work-related costs [10].

Abdominal symptoms after LC are often summarized as “postcholecystectomy syndrome.” However, postcholecystectomy syndrome is an arbitrary term that loosely describes the presence of symptoms after LC and consists of many persistent and incident symptoms [11–15]. In order to help patients with abdominal symptoms after LC, a specific diagnosis or etiology of the complaints is needed to provide targeted treatment. Therefore, this systematic review is aimed at providing an overview of the literature on etiologies of abdominal symptoms after LC and ultimately to assist clinicians in identifying the cause of patients' symptoms after LC and optimize treatment.

## 2. Methods

The PRISMA guideline (Preferred Reporting Items for Systematic Reviews and Meta-Analyses) was used to perform this systematic review [16].

### 2.1. Search Strategy

A systematic literature search was conducted in the electronic databases of MEDLINE (1946–June 2018), Web of Science (1945–June 2018), and EMBASE (1980–June 2018). The search was performed using a search strategy that included terms for “(postcholecystectomy) abdominal symptoms,” “cholecystectomy,” and “cholecystolithiasis” (the full search strategy is shown in Supplementary Table 1).

### 2.2. Study Selection

Two reviewers (C.L. and S.W.) independently screened the titles and abstracts of the identified articles to select potentially relevant studies. Studies on abdominal symptoms after LC in uncomplicated cholecystolithiasis patients ≥ 18 years, reporting at least one potential etiology for long-term symptoms, were eligible for inclusion. Long-term symptoms after LC were defined as any type of abdominal symptoms that were present at least four weeks after LC. Case reports, case series, editorials, and studies in a language other than English, Dutch, or German were excluded. Studies including patients after open cholecystectomy were excluded, as this does not reflect current surgical practice [17]. Discrepancies between the reviewers were resolved by discussion and consensus. In case of overlapping data, the most recent study with the largest cohort was included.

### 2.3. Data Extraction and Synthesis

Data were independently extracted by the two reviewers (C.L. and S.W.), using a predefined data extraction form. All described etiologies for long-term postcholecystectomy symptoms and the prevalence of these etiologies (if provided in the study) were extracted. Further extracted data included the following study characteristics: author, year of publication, country, study design, sample size, and follow-up period, and additional data on patients' age and gender, and long-term postcholecystectomy symptoms. Again, discrepancies between reviewers were resolved by discussion and consensus.

Subsequently, all etiologies were categorized as etiology for “persistent symptoms” or “incident symptoms” after LC and reported in subgroups per category. Persistent symptoms were defined as symptoms that are similar to patients' preoperative symptoms. Incident symptoms were defined as symptoms that were not present before LC. Primary outcomes of this review were the etiologies of persistent and incident symptoms after LC; the range in prevalence of each etiology in the included studies was reported.

## 3. Results

### 3.1. Selected Studies

**Figure 1:**
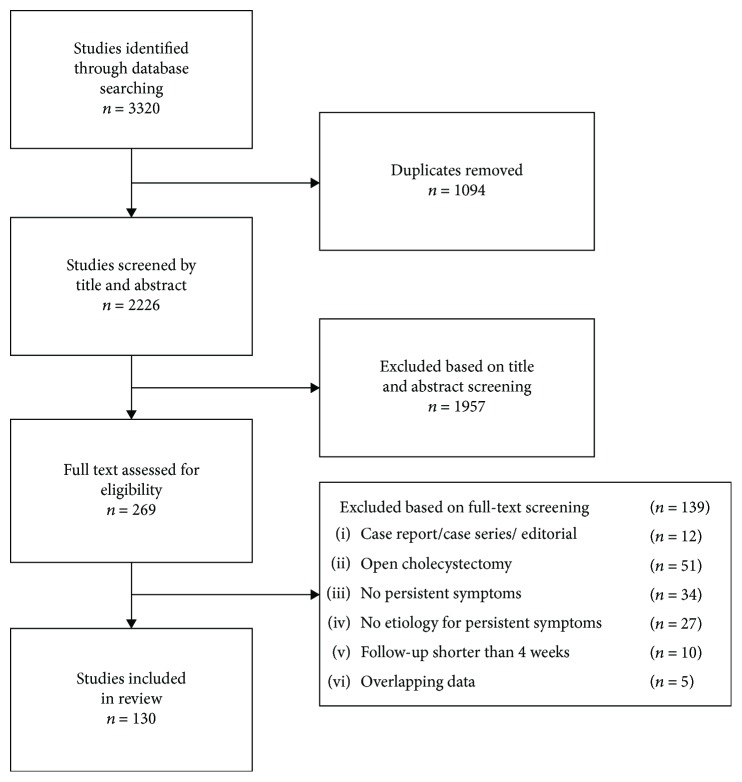


The search strategy identified 3320 articles. After removal of duplicates, titles and abstracts of 2226 articles were screened and 269 articles were selected for full-text evaluation. Finally, 130 articles were included in this review, as shown in Figure 1.

### 3.2. Study Characteristics

The included studies composed of 77 prospective cohort studies, 24 retrospective cohort studies, 20 reviews, five randomized controlled trials, and four systematic reviews. Most studies were performed in Europe and North America. The postoperative follow-up period in the included studies varied from four weeks to 18 years after LC. Full study characteristics are summarized in Table S2 in the supplementary files.

### 3.3. Reported Etiologies of Long-Term Symptoms after LC

The reviewed literature reported the following symptoms: biliary pain, pain attacks, continuous pain, pain related to food, functional dyspepsia, nausea, vomiting, abdominal bloating, reflux, diarrhea, constipation, functional bowel problems, fever, and jaundice.

**Figure 2:**
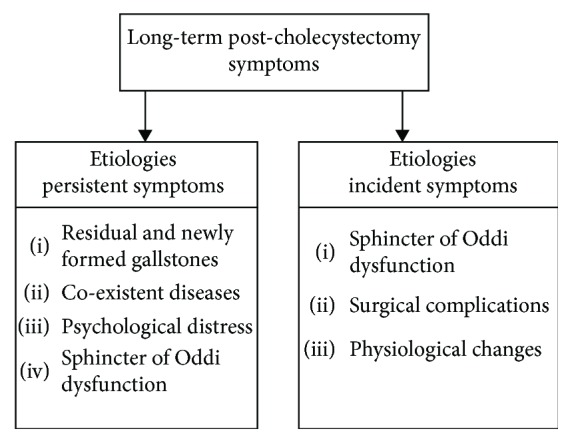


Persistent symptoms after LC were summarized into four subgroups: “residual and newly formed gallstones,” “coexistent diseases,” “psychological distress,” and “sphincter of Oddi dysfunction.” Three subgroups for etiologies of incident symptoms after LC were established: “sphincter of Oddi dysfunction,” “surgical complications,” and “physiological changes” (Figure 2). Sphincter of Oddi dysfunction (SOD) can cause persistent symptoms; however, most often it arises after LC. The etiologies reported per included study and if provided the percentage of patients with a certain etiology as cause for symptoms after LC are summarized in Table S2.

## 4. Persistent Symptoms

### 4.1. Residual and Newly Formed Gallstones

Forty-one studies reported residual or newly formed gallstones as the etiology for long-term persistent abdominal symptoms after LC. A total of 23 studies provided the prevalence of residual and newly formed gallstones as the cause for symptoms, ranging from 0.2% to 23%. Residual stones are most commonly diagnosed as retained common bile duct stones (choledocholithiasis), stones, or sludge in a cystic duct remnant or stones within the remnant gallbladder due to a subtotal cholecystectomy in difficult surgical cases. Residual stones in the cystic duct or gallbladder remnant can result in recurrent biliary colics [18–22]. Usually, these symptoms are self-limiting. Choledocholithiasis after LC is associated with epigastric pain, elevated ALT and AST levels, and sometimes jaundice [22–24]. Additional abdominal ultrasound might show a dilated common bile duct [19]. Moreover, new gallstones can be formed within the bile ducts or gallbladder remnants, after LC. Depending on the location in the biliary tract, symptoms will be similar to cystic duct or gallbladder remnant stones, or choledocholithiasis [25, 26].

### 4.2. Coexistent Diseases

Sixty-four studies reported coexistent diseases as the etiology for long-term persistent abdominal symptoms after LC. Eighteen studies provided the prevalence of coexistent diseases after LC ranging from 1% to 65%. Coexistent diseases in patients with gallstones are common and mainly nonbiliary: gastroesophageal reflux, peptic ulcer, hiatus hernia, gastritis, constipation, IBS, Anterior Cutaneous Nerve Entrapment Syndrome (ACNES), fatty liver disease, chronic obstructive pulmonary disease, or coronary artery disease [27, 28]. Preoperative distinction between symptoms caused by coexistent diseases and gallstones is challenging [29–32]. Misinterpretation of symptoms and suboptimal indication for LC will result in persistent symptoms after surgery [8, 9, 27, 30–36]. Even if the indication for LC was made correctly and the biliary symptoms are resolved, symptoms of a coexistent disease can become more prominent and considered as persistent symptoms after LC [29, 37].

### 4.3. Psychological Distress

Thirteen studies reported psychological distress as the etiology for long-term persistent abdominal symptoms after LC. None of these studies provided prevalence for psychological distress as cause for symptoms after LC. Several hypotheses exist on why psychologically distressed patients are more likely to experience persistent symptoms after LC. First, psychologically distressed patients tend to experience more functional gastrointestinal symptoms, which are not relieved by LC [38–40]. Secondly, psychological distress may induce visceral hyperalgesia that exacerbates subjective perception of pain both preoperatively and postoperatively [41]. Third, these patients are prone to experience somatization symptoms which may cause overreporting of symptoms [42]. Somatization symptoms are also less likely to be alleviated by surgery [39, 43]. Considering the different perceptions and interpretations, patients with psychological distress are more at risk for poor decision-making [38–40].

### 4.4. Sphincter of Oddi Dysfunction

Seventeen studies reported sphincter of Oddi dysfunction (SOD) as the etiology for long-term abdominal symptoms after LC. Prevalence of SOD after LC was reported in four studies and ranged from 3% to 40%. SOD mainly presents as right upper quadrant (biliary) pain and is not easily distinguished from symptomatic cholecystolithiasis, irritable bowel syndrome, or functional dyspepsia [44]. If SOD symptoms have been incorrectly attributed to gallstones, symptoms will persist after LC [15, 45, 46]. However, SOD most often commences after LC as incident symptoms, in which case interrupted neural pathways between the duodenum, gallbladder, and sphincter of Oddi after surgery lead to sphincter of Oddi spasms or SOD [47–50]. SOD can be divided in three types: type I (biliary pain, abnormal liver tests, and dilated bile duct), type II (biliary pain and abnormal liver tests or dilated bile duct), and type III (only biliary pain) [51, 52].

## 5. Incident Symptoms

### 5.1. Surgical Complications

Twenty-one studies reported surgical complications as the etiology for long-term symptoms after LC. Prevalence of long-term symptoms after LC caused by surgical complications was reported in eight studies, ranging from 1% to 3%. Bile duct injury is the most feared surgical complication [53, 54]. Patients can develop upper abdominal pain with jaundice, fever, and possibly sepsis [48]. Even if the bile duct injury is treated with surgical or endoscopic intervention, strictures or leakages can result in long-term symptoms of pain and biliary obstruction [15, 53, 55].

Spillage of gallstones into the peritoneal cavity is another complication associated with long-term postoperative pain, which can lead to abscesses, general peritonitis, adhesions, and fistulae, even several years after surgery [56–59]. However, the majority of dropped gallstones remain clinically silent [58].

Pain or discomfort due to late postoperative complications can arise from infections, wound healing problems, or a trocar site hernia [40, 60–67].

### 5.2. Physiological Changes

Thirty-nine studies reported physiological changes after surgery as the etiology for incident abdominal symptoms after LC. The prevalence of physiological changes after LC was described in 17 studies, ranging from 16% to 58%. Long-term effects of LC on bile acid metabolism were reported in several studies [61, 68–70]. Prior to LC, bile acids are stored in the gallbladder and bile acids are released in the duodenum by meal-induced intermittent contractions. LC results in the loss of reservoir function of the gallbladder and an altered bile metabolism. The pathophysiology of increased bile flow has not been completely clarified. However, the continuous flow of bile acids into the duodenum attributes to increased duodenal-gastric reflux and can cause symptoms of dyspepsia and an elevated risk of gastritis [49, 71–73]. Decreased esophageal sphincter pressure after LC may further attribute to dyspepsia and gastritis symptoms [61, 66, 74].

The reduced bile salt pool after LC could also induce subclinical fat malabsorption and result in diarrhea. The constant presence of bile acids in the gut, which promotes secretion and motility, could additionally result in a shortened whole gut transit time, contributing to postoperative diarrhea and flatulence [49, 68, 69].

### 5.3. Others

Fifteen studies reported various other etiologies for incident long-term abdominal symptoms after LC. Changed dietary intake, mainly waiving preoperative dietary restrictions, or physical inactivity may attribute to symptoms after LC [61, 75].

## 6. Discussion

**Figure 3:**
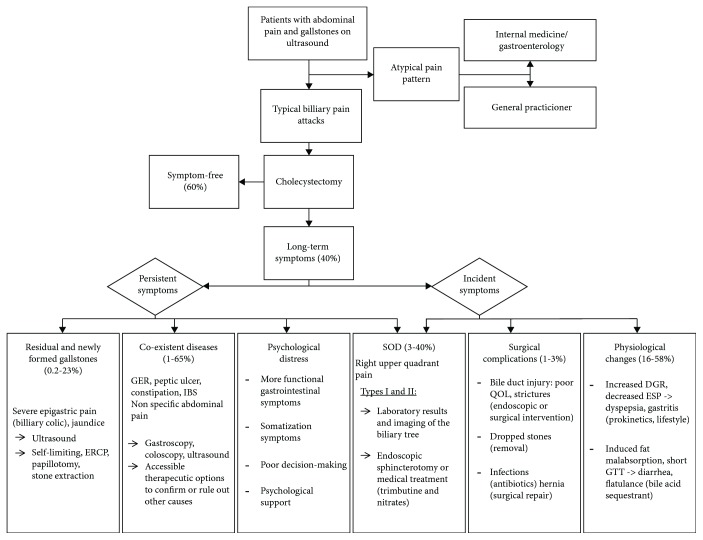


This systematic review provides a qualitative overview of etiologies of long-term abdominal symptoms after LC. Most symptoms after LC seem to be caused by coexistent diseases and physiological changes due to LC. Based on the etiologies of persistent and incident symptoms after LC provided in this review, we constructed a decision tree to help clinicians identify the cause of long-term symptoms after LC and optimize treatment for these patients (Figure 3).

“Postcholecystectomy syndrome” is a collective term for all symptoms after LC. This general term is not an adequate diagnosis [76–78], as multiple etiologies requiring distinct treatments may cause “postcholecystectomy syndrome.” Moreover, some symptoms are not even related to LC itself. To establish the cause of long-term symptoms after LC and decide on the proper treatment to alleviate symptoms, the underlying etiology of symptoms should be pursued.

Previous reviews divided all causes for symptoms after LC into organ systems [13] (e.g., biliary causes, pancreatic causes, other gastrointestinal disorders, or extraintestinal disorders) or listed all diagnoses individually (e.g., peptic ulcer disease, hiatus hernia, gastroesophageal reflux, residual stones, strictures, and SOD) [76]. The latter is a review with a limited search reach and only 21 included articles [76]. In this review, we categorized long-term postoperative symptoms as persistent or incident symptoms after LC, thereby providing a first step in deducting the causes for long-term symptoms. If the persistent or incident nature of the symptoms is established, the categories and subgroups presented in this review are a tool for clinicians in the assessment of long-term postcholecystectomy symptoms (Figure 3).

We established that most persisting symptoms are likely to be caused by coexisting diseases; often, these will be nonbiliary symptoms. Detailed anamnesis and tailored diagnostic tests (such as ultrasound, gastroscopy, and colonoscopy) will provide insight in the presence of (functional) abdominal disorders. Accessible therapeutic options should confirm or rule out the diagnosis, for instance, with a test treatment with antacids or laxatives [26, 32, 35, 36, 79].

Persisting biliary pain will mainly be caused by newly formed or residual stones or SOD. These conditions can be diagnosed using abdominal or endoscopic ultrasound. Gallstones will most often be present in the CBD and can be treated by ERCP with papillotomy and stone extraction. SOD type I and II can be distinguished from other disorders by laboratory results, imaging of the biliary tree, and elevated sphincter pressure at manometry. SOD type III is difficult to distinguish from other gastrointestinal disorders, as the only criterion is biliary pain. Some literature recommends endoscopic sphincterotomy to treat SOD [15, 45]; however, recently published long-term results of the EPISOD study show that in type III SOD, endoscopic sphincterotomy was not more successful compared to sham intervention in patients with postcholecystectomy SOD type III [80, 81]. Another study recommends medical treatment, trimebutine, and nitrates taken sublingually, as success rates are similar with endoscopic sphincterotomy [82].

Newly formed symptoms will often start shortly after LC but can persist and become a long-term problem. Patients with surgical complications should therefore be monitored at the outpatient clinic to obviate persisting symptoms, and surgical, endoscopic, or medical treatment can be started timely (e.g., surgical or endoscopic intervention with a stent or dilatation for bile duct injury or antibiotics for (intra-abdominal) infections). Most incident symptoms will however be physiological. Patients with new reflux symptoms after LC (due to physiological changes in bile secretion and metabolism) can be pragmatically treated with lifestyle changes, drugs that reduce the secretion of gastric acids, prokinetic drugs, or drugs that reduce the relaxations of the esophageal sphincter, to reduce reflux and relieve symptoms. Patients with (invalidating) chronic diarrhea can be treated with a bile acid sequestrant like cholestyramine, colestipol, or colesevelam [83].

Although this study provides tools to establish and treat symptoms, of course, preventing postoperative symptoms is preferable. A prospective study showed that 56% of patients need additional health care and medical costs and costs for sick leave were approximately $916 per year per patients [10]. Improved patient selection and preoperative work-up for a LC could prevent persistent abdominal symptoms and costs.

In patients with nonspecific gallstone symptoms, the preoperative diagnostic trajectory should focus on confirming or ruling out other causes of upper abdominal symptoms and considering alternative or concomitant therapeutic options. Our research group is currently performing a multicenter prospective study (Dutch Trial Register: NTR7307) to identify the prevalence of functional gastrointestinal disorders (FGID) in patients with gallstones. Current literature suggests prevalence of up to 60% [26, 39, 84, 85]. If such high prevalence is accurate, a large part of persistent symptoms after LC could be explained by coexistent FGID and treatment to prevent persistent symptoms could be initiated prior to surgery. A second prospective study (NTR7267) focuses on establishing the abdominal symptoms for appropriate indication of LC to prevent persistent symptoms caused by wrong surgical indication.

Additionally, shared decision-making and increased influence in choosing their preferred treatment may result in improved physical outcomes and less distress. This is illustrated for psychologically distressed patients [42] but may very well apply for other patients. Furthermore, we should consider that symptoms before and after LC may be present as part of the metabolic syndrome [86–88]. The metabolic syndrome is described as the underlying disorder for gallstones by abnormalities of insulin resistance, resulting in increased biliary cholesterol synthesis and gallstone formation [86]. LC is aimed at treating the gallstone symptoms, but the lifestyle and other comorbidities associated with the metabolic problem remain untreated. Incorporation of lifestyle changes and treatment of other aspects of the metabolic syndrome could reduce postoperative symptoms in this patient category.

The present review comes with strengths and limitations. Strengthening our study are the broad search and wide inclusion criteria to identify all possible etiologies of long-term abdominal symptoms after LC. Articles on open cholecystectomy were excluded, to prevent bias by etiologies or prevalence (such as higher surgical complications) inherent to the open aspects of the surgery, not reflecting current surgical practice. Additionally, differentiation between incident and persistent symptoms and descriptions of subgroups of etiologies were made to improve clinical applicability of the results. Ultimately, a clinical guidance was provided for physicians in the diagnostics and treatments of patients with symptoms after LC.

Limitations include the large heterogeneity of the included studies and subsequent inability to perform a quality assessment. As only a limited number of included studies reported the prevalence of described etiologies, we could not provide a meta-analysis. Subsequently, we were only able to provide the range of prevalence of the different etiologies to illustrate which etiologies are more and less common.

## 7. Conclusion

Postcholecystectomy symptoms have multiple etiologies and can be divided into persistent and incident symptoms. Most symptoms seem to be caused by coexistent diseases and physiological changes due to LC. Although treatment is available for most causes of persistent symptoms after gallbladder removal, optimized indication for surgery remains key.

## Supplementary Materials

Supplementary Materials Table S1: search strategy. Table S2: study characteristics of included studies.
