# Supplementary material for: Etiologies of Long-Term Postcholecystectomy Symptoms: A Systematic Review
Source: Gastroenterol Res Pract. 2019 Apr 14;2019:4278373. doi: 10.1155/2019/4278373 (PMC6487117; doi:10.1155/2019/4278373)
Supplement: Supplementary Materials — Table S1: search strategy. Table S2: study characteristics of included studies. [file 4278373.f1.docx]

**SUPPLEMENTARY FILES**

**Table S1. Search strategy**

|  | MEDLINE | Web of Science | EMBASE |
| --- | --- | --- | --- |
| *Abdominal symptoms* | Abdominal pain[MeSH] **OR** dyspepsia[MesH] **OR** postcholecystectomy syndrome[MeSH] **OR** abdominal pain[tiab] **OR** abdominal complaints[tiab] **OR** abdominal symptoms[tiab] **OR** gastrointestinal pain[tiab] **OR** gastrointestinal complaints[tiab] **OR** gastrointestinal symptoms[tiab] **OR** gastro-intestinal complaints[tiab] **OR** gastro-intestinal pain[tiab] **OR** gastro-intestinal symptoms[tiab] **OR** gastro intestinal complaints[tiab] **OR** gastro intestinal pain[tiab] **OR** gastro intestinal symptoms[tiab] **OR** post-cholecystectomy syndrome[tiab] **OR** post-cholecystectomy symptoms[tiab] **OR** post-cholecystectomy complaints[tiab] **OR** post-cholecystectomy pain[tiab] **OR** postcholecystectomy syndrome[tiab] **OR** postcholecystectomy symptoms[tiab] **OR** postcholecystectomy complaints[tiab] **OR** postcholecystectomy pain[tiab] **OR** post cholecystectomy syndrome[tiab] **OR** post cholecystectomy symptoms[tiab] **OR** post cholecystectomy complaints[tiab] **OR** post cholecystectomy pain[tiab] **OR** post-cholecystectomy syndrome[tiab] **OR** postcholecystectomy syndrome[tiab] **OR** post cholecystectomy syndrome [tiab] **OR** dyspepsia[tiab] **OR** biliary pain[tiab] **OR** colic pain[tiab] **OR** functional pain[tiab] **OR** functional complaints[tiab] **OR** metabolic syndrome[tiab] | TS=abdominal pain **OR** abdominal complaints **OR** abdominal symptoms **OR** gastrointestinal pain **OR** gastrointestinal complaints **OR** gastrointestinal symptoms **OR** gastro-intestinal complaints **OR** gastro-intestinal pain **OR** gastro-intestinal symptoms **OR** gastro intestinal complaints **OR** gastro intestinal pain **OR** gastro intestinal symptoms **OR** post-cholecystectomy syndrome **OR** post-cholecystectomy symptoms **OR** post-cholecystectomy complaints **OR** post-cholecystectomy pain **OR** postcholecystectomy syndrome **OR** postcholecystectomy symptoms **OR** postcholecystectomy complaints **OR** postcholecystectomy pain **OR** post cholecystectomy syndrome **OR** post cholecystectomy symptoms **OR** post cholecystectomy complaints **OR** post cholecystectomy pain **OR** post-cholecystectomy syndrome **OR** postcholecystectomy syndrome **OR** post cholecystectomy syndrome **OR** dyspepsia **OR** biliary pain **OR** colic pain **OR** functional pain **OR** functional complaints **OR** metabolic syndrome | Abdominal pain/ **OR** dyspepsia/ **OR** postcholecystectomy syndrome/ **OR** gastrointestinal symptom/ **OR** biliary tract pain/ **OR** (abdominal pain **OR** abdominal complaints **OR** abdominal symptoms **OR** gastrointestinal pain **OR** gastrointestinal complaints **OR** gastrointestinal symptoms **OR** gastro-intestinal complaints **OR** gastro-intestinal pain **OR** gastro-intestinal symptoms **OR** gastro intestinal complaints **OR** gastro intestinal pain **OR** gastro intestinal symptoms **OR** post-cholecystectomy syndrome **OR** post-cholecystectomy symptoms **OR** post-cholecystectomy complaints **OR** post-cholecystectomy pain **OR** postcholecystectomy syndrome **OR** postcholecystectomy symptoms **OR** postcholecystectomy complaints **OR** postcholecystectomy pain **OR** post cholecystectomy syndrome **OR** post cholecystectomy symptoms **OR** post cholecystectomy complaints **OR** post cholecystectomy pain **OR** post-cholecystectomy syndrome **OR** postcholecystectomy syndrome **OR** post cholecystectomy syndrome **OR** dyspepsia **OR** biliary pain **OR** colic pain **OR** functional pain OR functional complaints **OR** metabolic syndrome).ti,ab,kw. |
| **AND** |  |  |  |
| *Cholecystectomy* | Laparoscopic cholecystectomy[MeSH Terms] **OR** Cholecystectomy[MeSH Terms] **OR** Cholecystectom*[tiab] | TS=Cholecystectom* | Laparoscopic cholecystectomy/ **OR** Cholecystectomy/ **OR** (Cholecystectomy*).ti,ab,kw. |
| **AND** |  |  |  |
| *Cholecystolithiasis* | Cholecystolithiasis[Mesh] **OR** Gallstones[Mesh] **OR** Cholecystolithiasis [tiab] **OR** Gallstone[tiab] **OR** Gallstones[tiab] | TS=Gallstone **OR** Gallstones **OR** Cholecystolithiasis | Gallstone/ **OR** (Gallstone **OR** Gallstones **OR** Cholecystolithiasis) .ti,ab,kw. |
| *Search total* | **829** | **877** | **1614** |

Full search strategy in the electronic databases of MEDLINE (1946 – June 2018), Web of Science (1945 – June 2018) and EMBASE (1980 – June 2018).

**Table S2. Study characteristics of included studies**

| **Author** | **Year** | **Country** | **Study design** | **Sample size (n)** | **Females (%)** | **Age in years (mean/ range)** | **Post-operative follow-up period** | **Etiologies (prevalence‡)** |
| --- | --- | --- | --- | --- | --- | --- | --- | --- |
| vanSonnenberg | 1993 | USA | Prospective cohort | 21 | 76.2 | (21-72) | 1.5 year | Complications (NA), Gallstones (NA) |
| Qureshi | 1993 | Ireland | Retrospective cohort | 100 | 77.0 | 47 | 1 year | Physiological (NA), Gallstones (2%), Co-existent diseases (NA) |
| Vander Velpen^61^ | 1993 | Scotland | Prospective cohort | 124 | 62.9 | 62 (29-87) | 6 months | Physiological (18%), Psychological (NA), Other (7%) |
| Schwery | 1993 | Swiss | Retrospective cohort | 49 | 65.3 | 64 (22-88) | 1-3 years | Gallstones (56%*), Complications (NA) |
| Wilson^63^ | 1993 | UK | Retrospective cohort | NR | NR | 53 | 1 year | Other (NA) |
| Metzger | 1993 | Swiss | Prospective cohort | 116 | 59.5 | 54 | 1 year | Co-existent diseases (3%) |
| Abu Farsakh | 1995 | Jordan | Prospective cohort | 56 | 85.7 | 44 (22-70) | 3-30 months | Physiological (50%) |
| Jones^69^ | 1995 | USA | Retrospective cohort | 225 | 76.9 | 47 (18-82) | 3.5 years | Physiological (NA), Co-existent diseases (NA), Gallstones (NA) |
| Schmitt | 1995 | USA | Retrospective cohort | 52 | 63.5 | 44 (16-93) | 2 years | Gallstones (31%^†^), Complications (16%^†^) |
| Niv^31^ | 1995 | Israel | Prospective cohort | 57 | 64.9 | 59 (10-90) | 1 year | Co-existent diseases (NA) |
| Ure^67^ | 1995 | Germany | Prospective cohort | 468 | 76.3 | 50 | 1-3 years | Co-existent diseases (NA), Gallstones (NA), Other (NA) |
| Fenster | 1995 | USA | Prospective cohort | 225 | 74.2 | 50 (12-79) | 3 months | Co-existent diseases (NA) |
| Buess^65^ | 1995 | Germany | Prospective cohort | 400 | NR | NR | 1-3 year | Complications (3%) |
| Lund^53^ | 1996 | USA | Review | NA | NA | NA | NA | Complications (NA), Gallstones (NA) |
| Kimura | 1996 | Japan | Prospective cohort | 110 | 54.5 | 53 | 1-3.5 years | Gallstones (NA) |
| Yoshitomi | 1996 | Japan | Prospective cohort | 22 | 77.3 | 51 (25-69) | 1 month | Physiological (NA) |
| Abu Farsakh | 1996 | Jordan | Prospective cohort | 30 | 66.7 | 44 (22-70) | 2 years | Physiological (17%), Co-existent diseases (43%) |
| Gui | 1996 | UK | Prospective cohort | 92 | 77.8 | 51 (24-84) | 1-7 years | Co-existent diseases (NA) |
| Sondenaa | 1997 | Norway | Prospective cohort | 184 | 72.3 | 53 (20-79) | 1.5-4 years | Co-existent diseases (NA) |
| Peterli^64^ | 1998 | Swiss | Prospective cohort | 253 | 76.3 | 54 (18-90) | 1 year | Gallstones (1%), Other (4%), Co-existent diseases (30%) |
| Denzil | 1999 | Mexico | Retrospective cohort | 287 | 87.8 | (20-87) | 1 month | Complications (1%), Gallstones (0.3%) |
| Cafarella^59^ | 1999 | USA | Review | NA | NA | NA | NA | Complications (NA) |
| Victorzon^62^ | 1999 | Finland | Prospective cohort | 300 | 75% | 51 | 2 years | Physiological (NA), Gallstones (0.4%), Co-existent diseases (NA) |
| Borly^41^ | 1999 | Denmark | Prospective cohort | 80 | 88.8 | 45 (20-81) | 1 year | Co-existent diseases (NA), Psychological (NA) |
| van der Velden^30^ | 1999 | the Netherlands | Retrospective cohort | 133 | 73.7 | 50 (18-80) | 1-4.5 years | Co-existent diseases (NA) |
| Middelfart | 1999 | Denmark | Prospective cohort | 10 | 90.0 | 51 (29-66) | 3-9 months | SOD (NA) |
| **Author** | **Year** | **Country** | **Study design** | **Sample size (n)** | **Females (%)** | **Age in years (mean/ range)** | **Post-operative follow-up period** | **Etiologies (prevalence‡)** |
| Corazziari^50^ | 1999 | Australia | Review | NA | NA | NA | NA | SOD (NA) |
| Manifold | 2000 | UK | Prospective cohort | 17 | 64.7 | 53 (32-74) | 3 months | Co-existent diseases (NA), other (NA) |
| Weinert^74^ | 2000 | USA | Prospective cohort | 2481 | 77.8 | 48 (1-96) | 6 months | Physiological (28%), Psychological (NA), Co-existent diseases (NA) |
| Niranjan^66^ | 2000 | India | Prospective cohort | 113 | 79.6 | 41 (17-80) | 1.5 years | Physiological (20%) |
| Kennedy | 2000 | UK | Retrospective cohort | 3169 | 54.2 | NR | NR | Co-existent diseases (NA) |
| Mjaland^27^ | 2000 | Norway | Prospective cohort | 806 | 73.0 | 56 (18-91) | 3 months | Co-existent diseases (7%), Gallstones (2%) |
| Cicala^45^ | 2000 | Italy | Prospective cohort | 75 | 62.7 | 49(14-85) | NA | SOD (40%) |
| Diculescu^49^ | 2001 | Romania | Systematic Review | NA | NA | NA | NA | Physiological (20-30%), SOD (NA), Complications (1%) |
| Contracter | 2001 | Saudi-Arabia | Prospective cohort | 62 | 72.6 | 40 (16-80) | 2 days - 5 years | Gallstones (17%†) |
| Gronroos^23^ | 2001 | Finland | Retrospective cohort | 57 | 71.9 | 63 (22-84) | 1 month - 4 years | Gallstones (26†) |
| Fahlke^35^ | 2001 | Germany | Prospective cohort | 700 | NR | NR | 6 months | Co-existent diseases (NA), Gallstones (NA) |
| Yamaner^55^ | 2002 | Turkey | Prospective cohort | 161 | 64.3 | 59 (18-85) | 2 days – 4 years | Complications (33%†), Gallstones (53%†) |
| Carrilho-Ribeiro | 2002 | Portugal | Prospective cohort | 36 | 83.3 | 57 (43-69) | 8 years | Physiological (NA), Other (NA) |
| Ahmad^73^ | 2002 | India | Prospective cohort | 30 | NR | NR | NR | Physiological (23%) |
| Nakano^79^ | 2002 | USA | Prospective cohort | 25 | NR | NR | NR | Co-existent diseases (NA) |
| Quallich | 2002 | USA | Prospective cohort | 60 | 83.3 | 44 | NR | Co-existent diseases (NA), Gallstones (NA) |
| Mulvihill^54^ | 2003 | USA | Review | NA | NA | NA | NA | Complications (NA) |
| Berger^36^ | 2003 | the Netherlands | Systematic Review | NA | NA | NA | NA | Co-existent diseases (NA) |
| Lorusso^40^ | 2003 | Italy | Prospective cohort | 52 | 80.8 | 45 | 1 year | Psychological (NA) |
| Gad Elhak | 2004 | Egypt | Prospective cohort | 46 | 56.5 | 42 | 1 year | Physiological (17%) |
| Ros^60^ | 2004 | Sweden | RCT | 724 | NR | 51 | 1 year | Physiological (NA), Gallstones (NA), Co-existent diseases (NA), Other (NA) |
| Berger | 2004 | the Netherlands | Prospective cohort | 223 | 78.9 | 48 (17-81) | NA | Co-existent diseases (NA) |
| Stefaniek^42^ | 2004 | Poland | Retrospective cohort | 367 | NR | 53 | 1 year | Psychological (NA) , SOD (NA) |
| Barthet^47^ | 2004 | France | Review | NA | NA | NA | NA | SOD (NA) |
| Verthrus | 2005 | Norway | RCT | 263 | 81.4 | (21-88) | 5 year | Physiological (NA), Gallstones (3%), Other (NA) |
| Bisgaard | 2005 | Denmark | Prospective cohort | 150 | 86.0 | 41 (20-79) | 1 year | Other (5%) |
| Wald^52^ | 2005 | USA | Review | NA | NA | NA | NA | SOD (NA) |
| Berhane | 2006 | Norway | Prospective cohort | 220 | 73.6 | 47 (19-79) | NR | Co-existent diseases (NA) |
| **Author** | **Year** | **Country** | **Study design** | **Sample size (n)** | **Females (%)** | **Age in years (mean/ range)** | **Post-operative follow-up period** | **Etiologies (prevalence‡)** |
| Stawowy | 2006 | Denmark | Prospective cohort | 37 | 91.9 | 40 (20-65) | 12 weeks | Other (NA) |
| Kjaer | 2006 | Denmark | Prospective cohort | 25 | 96.0 | 48 (25-68) | 2-7 year | Other (NA) |
| Jorgensen | 2006 | Denmark | Prospective cohort | 115 | 65.2 | NR | 6-12 months | Psychological (NA) |
| Behar^44^ | 2006 | USA | Review | NA | NA | NA | NA | SOD (NA) |
| Halldestam^37^ | 2007 | Sweden | Prospective cohort | 200 | 80.5 | 47 (24-79) | 1 year | Co-existent diseases (NA) |
| Porcelli^38^ | 2007 | Canada | Prospective cohort | 52 | NR | NR | 1 year | Psychological (NA) |
| George^51^ | 2007 | USA | Review | NA | NA | NA | NA | SOD (NA) |
| Vignolo | 2008 | Brazil | Prospective cohort | 29 | 89.7 | 44 (17-77) | 6 months | Physiological (NA) |
| Fisher^70^ | 2008 | Australia | Prospective cohort | 98 | 63.3 | 58 | 6-12 months | Physiological (17%), Psychological (NA) |
| Mertens^26^ | 2008 | the Netherlands | Prospective cohort | 183 | 74.3 | 46 | 6 weeks | Co-existent diseases (57%) |
| McNally | 2008 | USA | Prospective cohort | 852 | 55.0 | 46 | 9-15 years | Co-existent disease (NA), Physiological (NA) |
| Okoro | 2008 | USA | RCT | 12 | 83.3 | (32-51) | 3 months | Gallstones (8%*), Co-existent diseases (39%*), SOD (3%*) |
| Shaffer | 2009 | Canada | Review | NA | NA | NA | NA | Physiological (NA), Co-existent diseases (NA), SOD (NA) |
| Amir | 2009 | Pakistan | Prospective cohort | 200 | 71.0 | 47 (14-92) | 6 months | Physiological (3%) |
| Shi | 2009 | Taiwan | Prospective cohort | 159 | 45.3 | 57 | 6 months | Co-existent diseases (NA) |
| Jaunoo | 2010 | UK | Review | NA | NA | NA | NA | Gallstones (NA), Physiological (NA), Co-existent diseases (NA), Complications (NA), SOD (NA) |
| Chowbey | 2010 | India | Retrospective cohort | 26 | NR | NR | 3-13 years | Gallstones (0.2%) |
| Dimitriou | 2010 | Germany | Retrospective cohort | 766 | NR | NR | NR | Co-existent diseases (16%) |
| Mertens | 2010 | the Netherlands | Prospective cohort | 172 | 76.2 | 48 | 6 months | Psychological (NA), Co-existent diseases (NA), Gallstones (NA), Physiological (NA) |
| Mertens^43^ | 2010 | the Netherlands | Prospective cohort | 133 | 75.9 | 47 | 6 weeks | Psychological (NA) |
| Sezeur^18^ | 2011 | France | Prospective cohort | 143 | 72.0 | 57 (20-85) | 2.5 years | Gallstones (15%) |
| Thistle^28^ | 2011 | USA | Prospective cohort | 1008 | 76.4 | 53 | 1 year | Co-existent diseases (NA) |
| Kirk | 2011 | Ireland | Prospective cohort | 112 | 87.5 | 53 (23-81) | 2 years | Co-existent diseases (NA) |
| Carraro^33^ | 2011 | Italy | Review | NA | NA | NA | NA | Co-existent diseases (NA) |
| Skalicky | 2011 | Slovenia | Prospective cohort | 80 | 56.3 | 60 (19-83) | 6 months | SOD (NA) |
| Uyanikoglu^71^ | 2012 | Turkey | Prospective cohort | 24 | 54.2 | 49 (28-74) | 3 months | Physiological (NA), Co-existent diseases (NA) |
| Atak | 2012 | Turkey | Prospective cohort | 85 | 76.5 | 45 | 6 months | Physiological (16%), Co-existent diseases (65%) |
| Aprea | 2012 | Italy | Prospective cohort | 31 | 58.1 | 75 (70-85) | 6 months | Physiological (58%) |
| **Author** | **Year** | **Country** | **Study design** | **Sample size (n)** | **Females (%)** | **Age in years (mean/ range)** | **Post-operative follow-up period** | **Etiologies (prevalence‡)** |
| Greenfield^48^ | 2012 | USA | Review | NA | NA | NA | NA | Gallstones (NA), SOD (NA), Complications (NA) |
| Lamberts | 2012 | the Netherlands | Systematic Review | NA | NA | NA | NA | Co-existent diseases (NA), Physiological (NA) |
| Schmidt | 2012 | Norway | Review | NA | NA | NA | NA | Co-existent diseases (NA), Physiological (NA) |
| Schmidt | 2012 | Norway | Prospective cohort | 153 | 79.7 | 48 (17-85) | 6 months | Co-existent diseases (NA) |
| Schonfels | 2012 | Germany | Retrospective cohort | 1915 | 72.2 | 60 | 8 years | Gallstones (2%) |
| Nayak^58^ | 2013 | USA | Review | NA | NA | NA | NA | Complications (NA) |
| Harju^32^ | 2013 | Finland | Prospective cohort | 127 | 80.3 | 49 (17-78) | 7-14 years | Co-existent diseases (38%) |
| Aspinen | 2013 | Finland | Prospective cohort | 48 | 79.2 | 43 (18-66) | 5 years | Co-existent diseases (NA) |
| Karmacharya | 2013 | Nepal | Prospective cohort | 96 | 87.5 | 43 (25-64) | 1 month | Co-existent diseases (10%) |
| Brown | 2013 | UK | Retrospective cohort | 100 | NR | NR | NR | Co-existent diseases (6%) |
| Pazouki^57^ | 2014 | Iran | Prospective cohort | 900 | 73.9 | 45 (31-84) | 1 year | Complications (2%) |
| Badretdinova | 2014 | Russia | Retrospective cohort | 47 | 96.6 | 59 | 8-12 years | Physiological (NA) |
| Rana | 2014 | India | Retrospective cohort | 128 | 69.5 | 47 | 6 months-15 years | Physiological (27%) |
| Ebied | 2014 | UK | Retrospective cohort | 640 | NR | NR | NR | Gallstones (1%), Co-existent diseases (1%), Other (1%) |
| Mohamadnejad | 2014 | Iran | Prospective cohort | 112 | NR | NR | 3 months | Gallstones (44%*), SOD (21%*) |
| Philips^20^ | 2014 | USA | Retrospective cohort | 12 | 58.3 | 38 (24-57) | 2 weeks - 14 years | Gallstones (NA), Complications (NA) |
| Kambal^21^ | 2014 | UK | Prospective cohort | 330 | 75.8 | 50 (16-88) | NR | Gallstones (23%) |
| Lamberts | 2014 | the Netherlands | Prospective cohort | 126 | 73.8 | 48 | 5 years | Co-existent diseases (NA) |
| Aspinen | 2014 | Finland | Prospective cohort | 78 | 88.5 | 43 (19-73) | 1 year | Co-existent diseases (NA) |
| Bielefeldt^34^ | 2014 | USA | Review | NA | NA | NA | NA | Co-existent diseases (NA), Gallstones (NA), SOD (NA), Psychological (NA) |
| Wanjura^29^ | 2014 | Sweden | Prospective cohort | 451 | 72.5 | 52 | 4 years | Co-existent diseases (NA) |
| Kim | 2014 | Korea | Prospective cohort | 34 | 52.9 | 45 | 6 months | Co-existent diseases (NA), Psychological (NA) |
| Gurusamy | 2014 | UK | Review | NA | NA | NA | NA | Co-existent diseases (NA) |
| Jorgensen^40^ | 2014 | Denmark | RCT | 120 | NR | 46 | 1 year | Complications (NA) |
| Ye^25^ | 2015 | China | Systematic Review | 2030 | NR | NR | NR | Gallstones (0.4%), Physiological (13%), Complications (NA) |
| Cox | 2015 | Australia | Retrospective cohort | 61 | 59.0 | 57 (25-84) | 3 days - 18 years | Gallstones (NA) |
| Chowbey^19^ | 2015 | India | Review | NA | NA | NA | NA | Gallstones (NA), Co-existent diseases (NA), Complications (NA) |
| Bennett^56^ | 2015 | USA | Review | NA | NA | NA | NA | Complications (NA) |
| **Author** | **Year** | **Country** | **Study design** | **Sample size (n)** | **Females (%)** | **Age in years (mean/ range)** | **Post-operative follow-up period** | **Etiologies (prevalence‡)** |
| Lamberts | 2015 | the Netherlands | Prospective cohort | 342 | 78.4 | 50 | 12 weeks | Co-existent diseases (NA) |
| Leontyev | 2015 | Russia | Prospective cohort | 53 | 78.4 | (24-69) | 1 year | SOD (34%) |
| Makarova | 2016 | Russia | Prospective cohort | 145 | NR | NR | 10 year | Co-existent diseases (47%) |
| Wanjura | 2016 | Sweden | Prospective cohort | 451 | 72.5 | 55 | 3-5.5 years | Physiological (NA), Co-existent diseases (NA) |
| Tsai | 2016 | Taiwan | Retrospective cohort | 20836 | 70.0 | NR | 5 years | Physiological (13%) |
| Kumar Saroj | 2016 | India | Retrospective cohort | 22 | 72.7 | 27-95 | 15 years | Gallstones (100%^±^) |
| Han^46^ | 2016 | South Korea | RCT | 138 | 74.7 | 50 | 3 months | Gallstones (NA), SOD (NA), Physiological (NA), Other (NA) |
| Zhang | 2016 | China | Retrospective cohort | 144 | 59.0 | 52 | 2-6 years | Co-existent diseases (2%), Gallstones (NA) |
| Altomare^68^ | 2017 | Italy | Review | NA | NA | NA | NA | Physiological (NA), Other (NA) |
| Del Grande | 2017 | Brazil | Retrospective cohort | 150 | NR | NR | NR | Physiological (35%) |
| Hani^22^ | 2017 | Jordan | Retrospective cohort | 16 | 75.0 | 44 (26-71) | 12 years | Gallstones (0.6%) |
| Kuzhanthaivelu | 2017 | India | Prospective cohort | 60 | 81.7 | 42 (19-75) | 1 year | Co-existent diseases (20%) |
| Shin^75^ | 2017 | Korea | Prospective cohort | 59 | 49.2 | 48 | 3 months | Other (46%), Physiological (NA), SOD (NA) |
| Talseth | 2017 | Norway | Prospective cohort | 534 | 74.2 | 56.4 | 6 years | Physiological (5-20%), Co-existent diseases (10%), Psychological (NA) |
| Wennmacker^9^ | 2017 | the Netherlands | Prospective cohort | 360 | 77.5 | 50 | 6 months | Co-existent diseases (NA) |
| Wennmacker | 2017 | the Netherlands | Prospective cohort | 124 | 83.9 | 52 | 3 years | Co-existent diseases (32%*), Gallstones (7%*), Complications (6%*) |
| Zhang | 2017 | China | Prospective cohort | 1714 | 66.1 | 54 (14-90) | 3 years | Co-existent diseases (NA), Physiological (NA) |
| Klimenko | 2018 | Ukraine | Prospective study | 136 | 79.4 | 49 (22-78) | NR | Gallstones (NA) |
| Lamberts | 2018 | the Netherlands | Review | NA | NA | NA | NA | Co-existent diseases (NA) |
| Shirah | 2018 | Saudi Arabia | Prospective cohort | 272 | 59.2 | 37 (28-550 | NR | Gallstones (4%), Complications (2%), SOD (0.9%), Co-existent diseases (9%), Physiological (NA) |
|  |  |  |  |  |  |  |  |  |

NR, not reported; NA, not applicable; SOD, Sphincter of Oddi Dysfunction
**‡**the prevalence of an etiology of patients with a laparoscopic cholecystectomy for gallstones
* the prevalence of an etiology of patients with persistent symptoms
† the prevalence of an etiology of patients with persistent symptoms and ERCP
± the prevalence of an etiology of patients with laparoscopic re-exploration
